# Profiles on the Orientation Discrimination Processing of Human Faces

**DOI:** 10.3390/ijerph17165772

**Published:** 2020-08-10

**Authors:** Carmen Moret-Tatay, Inmaculada Baixauli-Fortea, M. Dolores Grau-Sevilla

**Affiliations:** 1 Escuela de Doctorado, Universidad Católica de Valencia San Vicente Mártir, San Agustín 3, 46002 València, Spain; 2 Faculty of Psychology, Universidad Católica de Valencia San Vicente Mártir, Sede Padre Jofré, 46100 Burjassot, València, Spain; inmaculada.baixauli@ucv.es (I.B.-F.); lola.grau@ucv.es (M.D.G.-S.)

**Keywords:** face recognition, orientation discrimination, cluster analysis, orientation encoding of faces, Karolinska Directed Emotional Faces

## Abstract

Face recognition is a crucial subject for public health, as socialization is one of the main characteristics for full citizenship. However, good recognizers would be distinguished, not only by the number of faces they discriminate but also by the number of rejected stimuli as unfamiliar. When it comes to face recognition, it is important to remember that position, to some extent, would not entail a high cognitive cost, unlike other processes in similar areas of the brain. The aim of this paper was to examine participant’s recognition profiles according to face position. For this reason, a recognition task was carried out by employing the *Karolinska Directed Emotional Faces.* Reaction times and accuracy were employed as dependent variables and a cluster analysis was carried out. A total of two profiles were identified in participants’ performance, which differ in position in terms of reaction times but not accuracy. The results can be described as follows: first, it is possible to identify performance profiles in visual recognition of faces that differ in position in terms of reaction times, not accuracy; secondly, results suggest a bias towards the left. At the applied level, this could be of interest with a view to conducting training programs in face recognition.

## 1. Introduction

Faces represent a fundamental characteristic of our identity. Not surprisingly, it is the first part we would hide to avoid being recognized. We all have witnessed advances in facial biometrics in our environment for many fields, from security and health care systems to social networks. Even if this issue has become crucial in smart cities, many challenges still remain [1]. Among these, one should bear in mind that some face-recognition technology is able to do this process under not the most optimal conditions, e.g., even for people wearing masks to combat the spread of Covid-19 [2]. Current approaches have focused their attention on predictions through artificial intelligence. In this way, computer vision performance often employs algorithms as depicted in approaches such as the Convolutional Neural Network [3]. However, is this effect transferable to the cognitive abilities of human beings? In other words, are we able to recognize a face in complex situations?

The human brain is able to process a great number of characteristics belonging to a familiar face in a matter of milliseconds and without apparent cognitive cost [4]. This type of processing seems to be inherent to the human being and fundamental for multiple areas; among them, one of the most crucial ones would be socialization [5,6,7]. Of interest, this process occurs in the area intended for human face recognition, named as FFA (face form area), which has been described as one of the most specialized regions for facial recognition in the human visual system [8]. More precisely, the FFA is located in the fusiform gyrus, Brodmann area 37, and has also been related to other tasks: word recognition [9] and objects [10], among other processes. However, even if all these processes might be sharing the same brain areas [11], their nature is different, as, e.g., words have to be learned in comparison to face recognition [12]. Visual processing, as described in the literature, begins with the coding of the orientation of the local border in the primary visual cortex named V_1_ [13]. The responses of the neurons belonging to the V_1_ area oversee the preferred orientation, producing a maximum level of response. Some of the most relevant models in face recognition have focused on neural components [7,14,15]. Through anatomic-functional evidence, it is stipulated that the human brain would make use of different but connected cognitive processes related to aspects of the stimulus in terms of variance and invariance. Thus, the most consistent and least variant stimuli would be supported in areas such as the intraparietal groove (related to spatial attention) and the auditory cortex (such as prelexical perception for associated names, among others) [7,8,16]. On the other hand, the more abstract and variant aspects would make use of the amygdala and the limbic system to address aspects of emotional processing, and the anterior temporal zone for aspects of identity recognition [7,14,17].

There is undeniable consensus on some inherent aspects of face recognition in neurotypical patients across the life span. Newborns show a preference for the upper, as opposed to the lower, part of a face [18], suggesting that not only sensory properties but also structural characteristics are of interest. On the other hand, evidence has shed light on how facial recognition can be affected in older adults, for both detection [19] and identification proposes [20]. Although studies with clinical samples are of interest, at a more basal level, other studies seem to indicate that the maturation of specialized processing throughout the life span also depends on previous experiences [21]. In this way, several factors can interfere, such as the number of expositions and even the way that a stimulus is presented [22,23]. The aging process seems to be related to qualitative changes as well as quantitative changes in the perception of the face, which would be reflected in aspects such as processing components or reaction time [24]. Some progress has been made in understanding the molecular mechanism of face recognition. However, there is no single or simple answer for the rehabilitation approach in behavioral terms. Some research points to the role of cognitive strategies [25,26]. In this way, it has been described that good recognizers would be distinguished not only by the number of faces they recognize but also by the number of these stimuli that they can reject as unfamiliar [27].

When it comes to the role of stimulus on face recognition, it is important to remember that position, to some extent, would not entail a high cost, unlike other stimuli, such as written words [26]. Moreover, in our daily routine, it is common to constantly face different positions for a face in our environment. Hence, the identification of a human face may imply the recognition of the invariant structure of aspects in dynamic environments of our daily life [13]. The most ecological environments will be related to low viewing conditions [28], in terms of lighting [29] or distance [30], among others. One of the most interesting variants that can include all the variables described above is the movement or the position of presentation and a face. However, the scientific literature supports the effects of cultural configuration on visuospatial skills [31]. Habits such as reading have a strong influence on the cognitive system and can introduce spatial biases at both the perceptual and representational levels of a wide range of stimuli. Specifically, biases towards the left have been found in readers of French origin [32] and towards the right in readers of Hebrew [33]. This might be related to attentional issues, but different strategies might also be expected in a participant profile. Therefore, an analysis of the participants’ performance in face recognition is proposed. The starting point for evaluating the best participants, as marked by the literature, would be the ability to discard new and unfamiliar faces by choosing different position levels.

## 2. Methods

### 2.1. Participants

A total of 26 Spanish university students participated in this study. Therefore, a total of 13 men and 13 women volunteered to participate in the study, ranging from 18 to 23 years old. In order to participate, all participants gave written informed consent as described by the University ethics committee (UCV/2017-2018/31). G*Power 3 [34] was employed to examine effect size, f^2^ = 0.15, probability of error, α = 0.05, and sample size under a repeated measures design.

### 2.2. Materials

**Figure 1:**
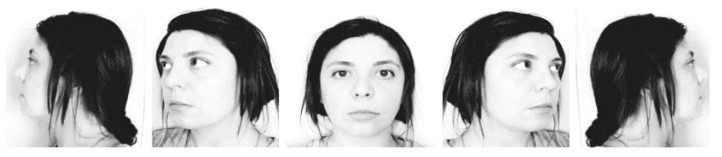
Due to copyright issues, exemplification of the position used from the Karolinska Directed Emotional Faces (KDEF) battery.

The Karolinska Directed Emotional Faces (KDEF) from the *Karolinska institutet* [35] was used. This consists of a total of 4900 images of facial expressions with different emotions and a total of 5 different positions: Central, Partial right, Right Profile, Partial Left and Left profile (see Figure 1 for an exemplification). A sample of 28 stimuli was chosen in order to exclusively select neutral expressions under these positions. Each stimulus was repeated several times under a repeated measures design. Therefore, for the present study, a total of 28 pictures (14 men and 14 women) matched in physical characteristics were selected. The total number of stimuli was 140. After a presentation block, participants were instructed to identify the previous stimuli and discard the novel ones. A Windows operating system computer was used with the free experimental DMDX software [36].

### 2.3. Procedure

The experiment consisted of two phases, a first called “presentation” with 24 photographs that appeared at random. After 5 min, the participants passed the second phase called “recognition”, where the previous stimuli appeared plus another 24 (48 in total). In this phase, the participants had to press the green key (M) if they recognized the image of the previous block and press the letter Z, or red, if they considered the image novel. Each session lasted approximately 15 min.

### 2.4. Design and Data Analysis

As mentioned before, this was an experimental design under repeated measures. This approach was selected as it reduces the variance of estimates in comparison with other designs, such as between subjects’ ones. Moreover, all participants came across all conditions, allowing statistical inference to be made with fewer subjects in comparison with other designs. In order to know the response profiles in the discarding of new information, a cluster analysis was performed, which, as a multivariate technique, seeks to group elements (or variables) to achieve maximum homogeneity in each group, as well as the greatest differences between them. One should bear in mind that cluster analysis has enabled the formation of homogeneous groups within multiple fields of cognitive science and public health [37,38]. For example, studies have developed dendrograms from the hierarchy clustering analysis based on the strength of functional connectivity among the face-selective specified regions of interest (or ROI’s) when the participants performed a face recognition task [39]. This procedure was similar to the previous literature for small samples [38,40]. Data were analyzed using SPSS IBM statistical software for Windows version 23.0 (IBM Corp., Armonk, NY, USA). Data were checked for multicollinearity and multivariate outliers. In addition, the Kolmogorov–Smirnov test was used to verify that the scores on the variables had a normal distribution. Cluster analysis was performed under the registration probability test based on the Schwarz Bayesian Inference Criterion (BIC). The proposed two-stage cluster analysis was replicated with a hierarchical cluster.

## 3. Results

First, face recognition was analyzed based on reaction time on the position stimuli. We were interested in the participants’ profile when discarding new information. In this way, a descriptive analysis was carried out. After examining the assumptions of interest, a cluster analysis was carried out. Descriptive statistics (response latencies and correct answers) were included in Table 1, as well as the increase in latencies (Δ) between the target and distractor stimuli.

The dependent variable of interest is the reaction time, as this is considered to reflect the cognitive architecture, and not surprisingly, is a star variable in the literature [41]. However, the RTs (reaction times) are drawn from positively skewed distributions; for this reason, extreme data were trimmed, as in previous literature [42]. Moreover, different assumptions were checked in terms of outliers and multicollinearity, and no more than 2% of the data were trimmed. The Kolmogorov–Smirnov test was used to examine whether the variables were normally distributed, *p* > 0.05. This was the same case for the Shapiro–Wilks normality test. Levene’s test indicated equal variances (all *p* > 0.05). The ANOVA on the distractor RTs showed that the target images were processed faster than the distractor images: F_(1.24)_ = 10.56; MSE = 36,479.79; *p* <0.001 η^2^ = 0.30. No position effect was found for response latencies, and no difference in efficacy across the hit rate (all *p* > 0.05).

Secondly, an exploratory two-stage cluster analysis was performed to identify the number of clusters in the participants on the distracting stimuli. Likewise, the Schwarz–Bayesian Inference Criterion (BIC) is shown in Table 2. We used it to select the lowest BIC value in the different estimated models, in this case for two clusters. After the analysis, 100% of the cases were included, the size ratio was optimal, with a value of 1.17. Two groups were formed with 46.2% and 53.8% of cases respectively. These two profiles were described as follows (see Table 2): a profile named G1 with slower and more conservative processing (n = 14) and a G2 profile with faster and more efficient processing (n = 12). In addition, depending on the values Δ, different response patterns can be described (see Table 3).

The participants’ sex was not related to the distribution of the new groups; moreover, it seemed to be distributed in a proportionate way, as the G1 was composed of seven men and seven women, while the G2 by six men and six women. As expected, the test χ^2^ did not depict sex differences for new clusters. In the analysis of these new groups, a non-parametric approach was chosen, as shown in Table 3.

As depicted in Table 4, the Mann–Whitney U test showed statistically significant differences for all conditions by cluster group. In addition to the Mann-Whitney U-test, jointly, the Vovk–Sellke indicators are offered to examine the maximum possible probability in favor of H₁ over H₀ [43]. This information was included to complement traditional *p*-value-based analyses, as suggested in the previous literature, through the use of probability [44]. All conditions were statistically significant for differences between groups. Also included was the Hodges–Lehmann’s estimate, with its confidence intervals, which would indicate the difference in the median between the two groups, and the bias-range correlation coefficient, which can be considered an effect size and is interpreted as the same as Pearson’s correlation coefficient [45].

As depicted in Table 5, the increments were addressed (Δ) following the previous procedure. This analysis attempts to shed light on differences in patterns by estimating the distance between target and distracting condition latencies. As well as the central position, considered more ecological, not presenting changes in the group increments, the differences between groups seem to mark a bias towards the left side, and the right side was the one that distinguishes the participants with better execution in the task. Finally, the proposed two-stage cluster analysis was replicated with a hierarchical cluster. The objective was to replicate the exploratory structure of the previous analysis.

**Figure 2:**
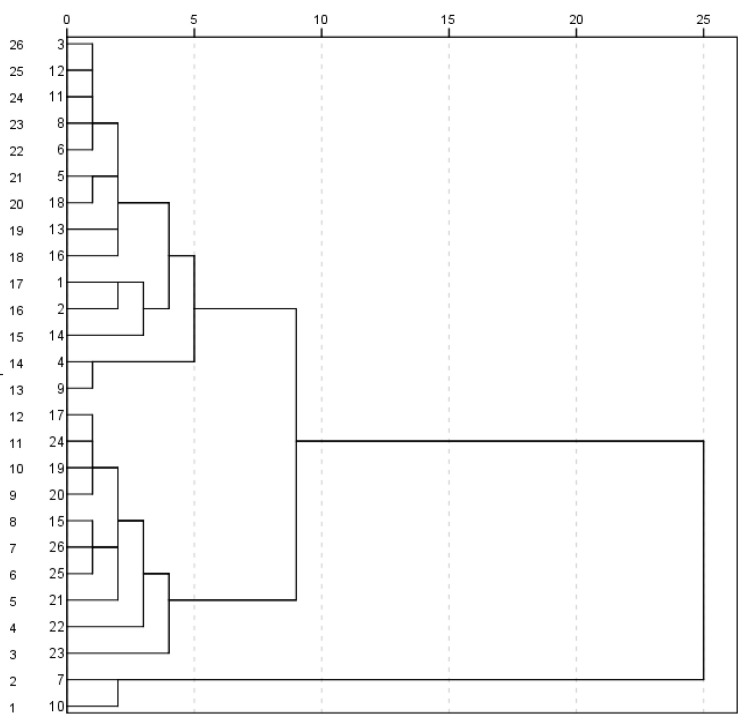
Tree diagram regarding the grouping of participants in a hierarchical cluster analysis.

In relation to the new hierarchical cluster, a representation of the suggested dendrogram is included, which replicates the previous structure. In Figure 2, this structure is presented in a tree diagram format that tries to illustrate the groupings of the participants. Two large groups are presented, consistent with the previous analysis, except for two subjects, who, although they are not within this subgroup, are closely linked.

**Figure 3:**
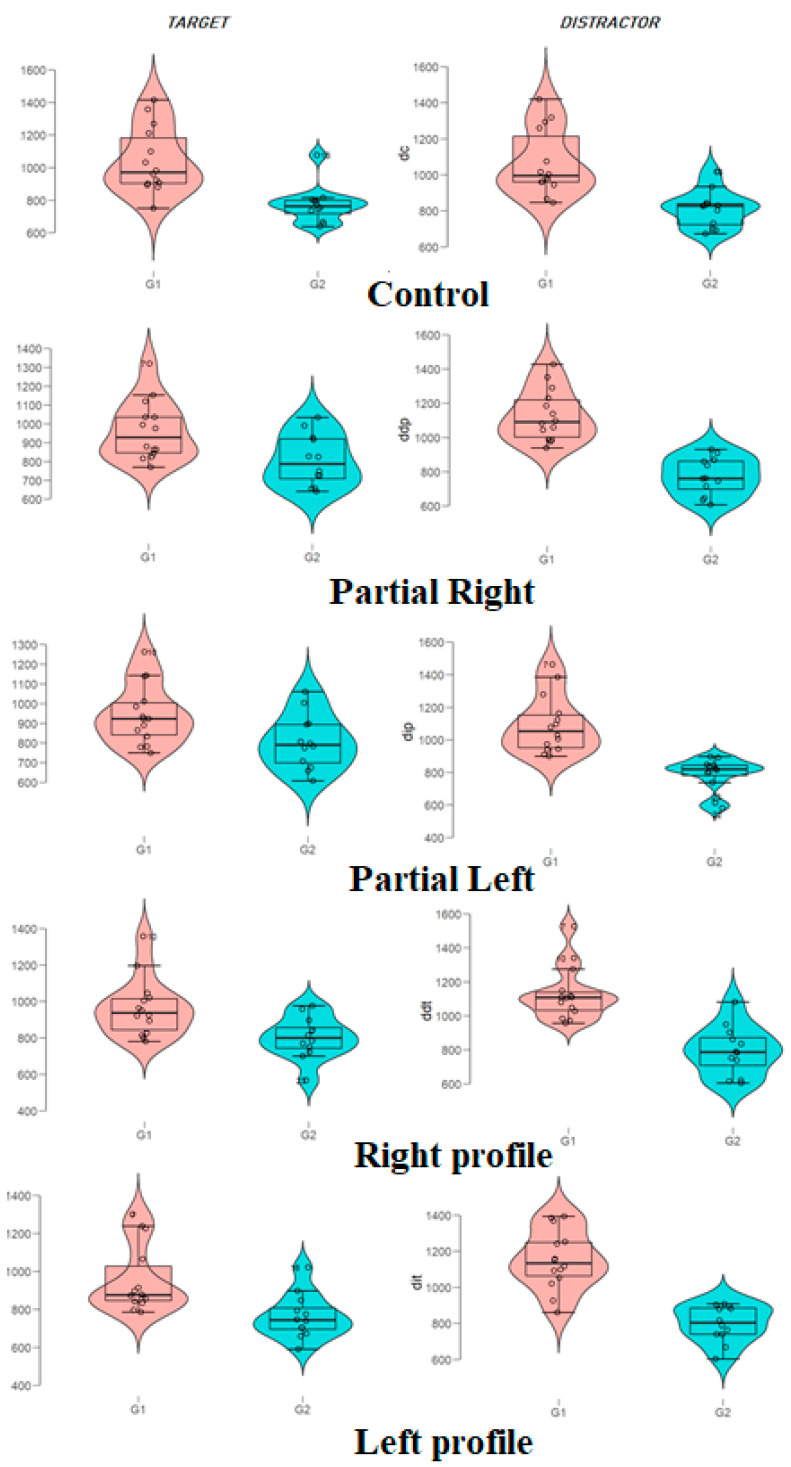
Box and whiskers diagram for the different conditions according to the cluster group.

Lastly, the box and whiskers diagrams for all experimental conditions by cluster group are shown in Figure 3. This representation allows us, in a very visual way, to know not only the central tendency statistics but also their relationship with the variability, which was markedly lower for group 2.

## 4. Discussion

Familiar versus unfamiliar recognition is of interest in the field of face processing, and its expert role in the visual system [22,46,47]. The holistic and features segmentation approaches have been described for both face and word recognition, suggesting the need for more research in terms of holistic versus feature perspective in the visual process as a continuum (and not only in isolated steps such as the perceptual, attentional or decisional one). The current results’ differences according to internal and external characteristics emphasize the role of participants’ strategies. In this way, the scientific literature supports the effects of cultural configuration on visuospatial skills [31]. It has been found that habits such as reading have a strong influence on the cognitive system and can introduce spatial biases at both the perceptual and representational levels of a wide range of stimuli. This aspect is of special interest when we must recognize a face, since, as a stimulus, unlike, for example, written words, it can appear in multiple positions and be processed, apparently without effort.

As mentioned before, a total of two profiles were found regarding their capacity to discard new information. The literature has described profiles of good recognizers, which would be distinguished not only by the number of faces they recognize but also by the number of these stimuli they are able to reject as unfamiliar [27]. The patterns between the two suggested groups showed differences between latencies or response times, so a more conservative pattern will be found for one of the profiles. From a qualitative approach, these results could support sensitivity to oblique information in the near horizontal range or biases [48,49] or preferences towards one of the orientations [32,33]. 

There are different limitations in this study. First, the sample of men and women is too small to examine possible gender differences. In addition, aspects such as the city of origin and its density have not been considered, which is stipulated to possibly moderate the recognition process. Future lines of research should conduct both direct and systematic replications over these issues and are expected to address these limitations. These should involve controlling the sex of participants and the density of their home populations. Moreover, the chosen stimuli were neutral, and therefore, future lines of research should include aspects such as the emotional valence of a face. As a first approach, a simpler manipulation has been proposed, and in this way, we hope it will serve for future manipulations that contemplate a greater variability gradient of the stimulus. Thus, the type of battery of stimuli used in this study, the Karolinska Directed Emotional Faces, is ideal for such a purpose as it was designed for emotional manipulations.

In sum, we consider these results of interests for training programs, where profiles can be of interest to understand participants’ strategies. At a theoretical level, this information is of interest to multiple fields, such as psychology or forensic medicine, by reducing errors within the psychology of testimony, and obviously, whether it is possible to determine face recognizer profiles would be of great interest for its social and personal consequences. At the applied level, this could be of interest not only with a view to conducting training programs in face recognition but also to better understand cognitive strategies. In this way, the literature has demonstrated the plastic capacity of the process, even in some clinical profiles [50,51,52]. Although the sample used is not a clinical one, we hope that future replications of the study can reach this level.

## 5. Conclusions

The aim of this paper was to examine recognition profiles, according to face position. This might shed light on differences in terms of strategies to deal with familiar versus unfamiliar stimuli. Therefore, a cluster analysis was carried out. The results can be described as follows: first, it is possible to identify performance profiles in visual recognition of faces that differ in position in terms of reaction times, not accuracy; secondly, results suggest a bias towards the left.

In sum, the main contributions and implications of the current work are listed as follows: First, to develop recognition profiles and encoding of facial stimuli in relation to the ability to discard new information. Secondly, to examine the role of facial stimulus invariance according to its orientation on human recognition, as described above. This type of contribution could offer a starting point in strategies based on the two points described in non-clinical participants.

## Figures and Tables

**Table 1:** Mean and SD (standard deviation), increases (Δ) and efficacy (%) in the fifth study.

	Target			Distractor			Δ	
	Mean	SD	Accuracy	Mean	SD	Accuracy	Mean	SD
Central	916.95	212.99	73	947.76	196.02	80	30.81	150.29
Partial right	891.12	164.97	78	964.48	222.65	77	73.37	154.19
Right Profile	889.37	160.33	75	974.39	225.47	75	85.02	148.27
Partial Left	880.43	159.18	75	953.07	209.97	82	72.64	164.29
Left profile	865.87	177.62	71	989.00	224.21	79	123.13	173.13

**Table 2:** Number of clusters based on the Schwarz Bayesian Inference Criterion (BIC).

Number	BIC	Δ BIC	Δ BIC Ratio	Distance Ratio
1	120.166			
2	111.049	−9.117	1.000	1.976
3	122.533	11.484	−1.260	4.279
4	150.185	27.651	−3.033	1.116
5	178.349	28.164	−3.089	1.571
6	208.118	29.770	−3.265	1.234
7	238.421	30.303	−3.324	1.174
8	269.062	30.640	−3.361	1.860
9	300.599	31.537	−3.459	1.031
10	332.168	31.569	−3.463	1.234
11	363.929	31.761	−3.484	1.015
12	395.702	31.773	−3.485	1.136
13	427.572	31.870	−3.496	1.070
14	459.488	31.916	−3.501	1.016
15	491.415	31.927	−3.502	1.066

**Table 3:** Mean and SD (standard deviation), increments (Δ) and efficiency (%) in the clusters.

		**Target**			**Distractor**			**Δ**	
**Group**	**Position**	**Mean**	**SD**	**Accuracy**	**Mean**	**SD**	**Accuracy**	**Mean**	**SD**
G_1_ n = 14	Central	1041.65	199.82	74	1065.77	180.95	71	24.12	177.35
	Partial right	963.89	156.61	77	1128.42	150.15	74	164.53	113.43
	Right Profile	964.09	159.41	74	1128.62	157.13	69	164.53	124.86
	Partial Left	944.57	151.65	74	1091.98	176.81	78	147.41	139.73
	Left profile	954.26	175.91	69	1151.47	163.41	74	197.21	184.43
G_2_ n = 12	Central	771.47	114.17	71	810.08	101.11	89	38.62	118.49
	Partial right	806.21	135.13	80	773.22	109.12	81	−33.00	126.12
	Right Profile	802.21	113.99	75	794.46	144.12	82	−7.74	118.77
	Partial Left	805.60	138.06	76	791.02	100.23	88	−14.58	151.19
	Left profile	762.75	116.57	72	799.45	99.52	86	36.71	113.10

**Table 4:** Mann–Whitney U test for experimental conditions in the clusters.

						95% IC Hodges-Lehmann		
	Position	W	*p*	VS-MPR *	Hodges-Lehmann	Inferior	Superior	Rank-Biserial Correlation
Target	Central	153.0	<0.001	296.13	235.59	133.007	406.296	0.821
	Partial right	132.0	0.013	6.64	150.83	39.058	285.781	0.571
	Right Profile	134.0	0.009	8.59	137.71	42.708	245.354	0.595
	Partial Left	125.0	0.036	3.09	126.63	7.592	248.429	0.488
	Left profile	144.0	0.001	41.69	155.48	78.479	278.485	0.714
Distractor	Central	157.0	<0.001	900.44	228.16	131.955	376.916	0.869
	Partial right	168.0	<0.001	115,426.96	340.01	225.884	463.676	1.000
	Right Profile	162.0	<0.001	4939.10	324.96	204.327	444.413	0.929
	Partial Left	168.0	<0.001	115,426.96	269.16	151.331	413.728	1.000
	Left profile	163.0	<0.001	7512.37	349.44	232.391	485.385	0.940

Vovk–Sellke Ratio *

**Table 5:** Mann–Whitney U test for experimental conditions in the clusters increments (Δ).

					95% IC Hodges-Lehmann		
Position	W	*p*	VS-MPR	Hodges-Lehmann	Inferior	Superior	Rank-Biserial Correlation
Central	83.00	0.980	1.000	−2.095	−132.04	119.8	−0.012
Partial right	146.00	<0.001	61.221	208.146	105.96	286.3	0.738
Right profile	139.00	0.004	17.727	169.989	71.61	277.1	0.655
Partial Left	142.00	0.002	29.110	134.609	65.32	235.7	0.690
Left profile	131.00	0.015	5.886	147.011	50.28	304.4	0.560
